# Supplementary material for: Horizontally transferred cell-free chromatin particles function as autonomous ‘satellite genomes’ and vehicles for transposable elements within host cells
Source: eLife. 2025 Sep 15;13:RP103771. doi: 10.7554/eLife.103771 (PMC12435896; doi:10.7554/eLife.103771)
Supplement: Supplementary file 2. [file elife-103771-supp2.docx]

|  |  |  |  |  |
| --- | --- | --- | --- | --- |

**Supplementary File 2.**

Clinical and demographic information of cancer patients and healthy individuals who provided blood samples for isolation of cell-free chromatin particles.

**Cancer patients**

| **Age** | **Sex** | **Diagnosis** |
| --- | --- | --- |
| 25 years | Female | Breast cancer |
| 35 years | Male | Tongue cancer |
| 22 years | Male | B-ALL |
| 32 years | Male | CML |
| 38 years | Male | Buccal mucosa cancer |

B-ALL = B cell acute lymphocytic lymphoma; CML = chronic myeloid leukaemia

**Healthy individuals**

| **Age** | **Sex** |
| --- | --- |
| 34 years | Male |
| 29 years | Female |
| 22 years | Male |
| 22 years | Male |
| 27 years | Male |
