# Supplementary material for: Horizontally transferred cell-free chromatin particles function as autonomous ‘satellite genomes’ and vehicles for transposable elements within host cells
Source: eLife. 2025 Sep 15;13:RP103771. doi: 10.7554/eLife.103771 (PMC12435896; doi:10.7554/eLife.103771)
Supplement: Supplementary file 3. [file elife-103771-supp3.docx]

**Supplementary File 3.**

**Cell lines used in this study**

| **Sr. no.** | **Cell Line** | **Tissue Origin** | **Source** |
| --- | --- | --- | --- |
| 1. | NIH3T3 | Mouse embryonic lung fibroblast | ATCC (CRL-1658) |
| 2. | MDA-MB-231 | Human breast cancer | ATCC (HTB-26) |
| 3. | Vero | monkey kidney | ATCC (Vero-CCL-81) |
| 4. | Dolly | Female dog | Spontaneous malignant round cell tumour in a female dog developed at our institute’s animal facility |
| 5. | B/CMBA. OV | mouse ovary | ATCC (RRID: CVCL_5926) |
| 6. | HEK293 | Human embryonic kidney | ATCC (CRL-1573™) |
| 7. | MRC5 | Human lung fibroblast | ATCC (CCL-212) |
